# Research on Preparation and Properties of Carbon Fiber Reinforced Zinc-Based Aluminum Rich Alloy Composite

**DOI:** 10.3390/ma15031087

**Published:** 2022-01-30

**Authors:** Bin Zhong, Shuaibang Hu, Zhengyang Yu, Xuanxuan Qiang, Hui Yang

**Affiliations:** College of Mechanical Engineering, Xi’an University of Science and Technology, Xi’an 710054, China; h463054800@163.com (S.H.); yzy550891186@163.com (Z.Y.); qxx___123@163.com (X.Q.); yh1607783652@163.com (H.Y.)

**Keywords:** carbon fiber, zinc-based aluminum rich alloy, composite material, fiber surface metallization, friction and wear performance

## Abstract

Aiming at the problems of poor bonding between the carbon fiber and the metal matrix and the friction and wear performance of the composite material during the preparation of carbon fiber reinforced zinc-based aluminum rich alloy composites, the carbon fiber surface metallization process was studied. Taking ZA27 as the research object, a new type of zinc-based aluminum rich alloy composite material was prepared by using surface metallized chopped carbon fibers with different contents as reinforcement materials. The microscopic morphology, element distribution and phase composition of the surface metallized carbon fiber and composite materials were characterized, and the hardness and friction and wear properties of the composite materials were tested. The results show that: the surface metallization of carbon fiber effectively reduces the diffusion of carbon elements into the matrix material during the sintering process, and improves the interface bonding between the carbon fiber and the matrix material; Compared with ZA27 alloy, the hardness of 6vt% carbon fiber is increased by 29.6%, and the average friction coefficient and wear rate are reduced by about 18.4% and 96%, respectively, indicating that the carbon fiber reinforced zinc-based aluminum rich alloy composite material optimizes the friction and wear performance of traditional materials.

## 1. Introduction

Zinc-based aluminum rich alloy is a new non-ferrous metal material that has been widely used at home and abroad in recent decades [1,2]. Due to its low melting point, low density and excellent mechanical properties [3,4], it is widely used in coal, petroleum, aerospace and other fields [5,6]. With the development of social industry, people’ requirements of zinc-based aluminum rich alloy’s friction and wear performance are becoming higher and higher. Therefore, in order to further enhance the anti-friction and wear-resistant properties of zinc-based aluminum rich alloys, as anti-friction and wear-resistant materials, the research and development of new zinc-based aluminum rich alloy has important practical significance [7,8]. Compared with the currently widely used copper-based wear-resistant alloy, the zinc-based aluminum rich alloy ZA27 which is studied in this paper has higher strength and lower density [9], so it can play a more important role in the lightweight design of parts. In addition, the world has abundant zinc reserves and the price of zinc alloy is only about 1/3 of traditional copper alloys, which means using high aluminum zinc is becoming more economical [10].

In recent years, people have mainly studied the friction and wear properties of zinc-aluminum alloys from the aspects of alloying, heat treatment and modification treatment [11,12,13]. With the continuous emergence of high-performance materials such as carbon fiber, graphene, and polyethylene fiber, non-metallic materials have strengthened. The research on non-metallic materials reinforced metal matrix composites has received extensive attention from international researchers [14,15,16]. Carbon fiber is a new type of high-strength, high-elastic fiber material, in which the carbon content exceeds 95%. In addition, it has various advantages such us low density, light weight, low thermal expansion coefficient, low friction coefficient, high temperature resistance and good self-lubricating performance [17,18]. Carbon fiber has higher specific strength and specific elastic modulus than metal materials, and it has higher toughness and impact resistance than ceramics [19]. Mahavira Dhan et al. [20] prepared carbon fiber reinforced AA6061-based composite materials by using AA6061 as the base metal and uncoated continuous long-spool pitch-based carbon fibers as reinforcement materials through squeeze casting process which has showed the hardness and ultimate tensile strength of the composite materials. As the content of carbon fiber increases, the ductility decreases. Sha Jianjun et al. [21] prepared nickel/copper-coated carbon fiber reinforced aluminum matrix composites using extrusion infiltration technology, and they studied the interface wettability, microstructure and mechanical properties of the composites. The results showed that the compared with reinforced aluminum matrix composites and nickel/copper coating has significantly improved the wettability of the interface between carbon fiber and matrix material. In addition, it has effectively inhibits the interface reaction between carbon fiber and aluminum matrix. The yield strength, ultimate tensile strength and elastic modulus of copper-coated carbon fiber reinforced aluminum matrix composites are about 124 MPa, 140 MPa and 82 GPa, respectively. The yield strength, ultimate tensile strength and elastic modulus of nickel-coated carbon fiber reinforced aluminum matrix composites are, respectively, about 60 MPa, 70 MPa and 79 GPa, the yield strength and ultimate tensile strength of the copper-plated Cf/aluminum composite material is almost twice that of the nickel-plated Cf/aluminum composite material, and the mechanical properties of the two composite materials are much higher than that of pure aluminum. Song Yang et al. [22] prepared carbon fiber reinforced copper-based composite materials by using cold pressing and sintering technology. The study showed that 0.2wt% carbon fiber powder increased the hardness and density of the composite material. Compared with copper-based materials without carbon fiber powder, the under the same test parameters, the friction coefficient of carbon fiber reinforced copper matrix composites increased by 13.7%, and the amount of wear decreased by about 45%. However, with the increasing of carbon fiber powder’s content, the physical properties of composite materials showed an obvious downward trend.

Based on the above research, it can be concluded that the appropriate carbon fiber content can improve the mechanical properties and wear resistance of the metal matrix to a greater extent [23,24]. Currently, there aren’t any reports about the carbon fiber-reinforced zinc-based aluminum rich alloy prepared by SPS spark plasma sintering technology and the effect of carbon fiber on the microstructure and performance. Therefore, this article takes the zinc-based aluminum rich alloy ZA27 as the research object, and the content is 3vt%, 6vt%, 9vt% surface metallized chopped carbon fiber as a reinforcing material, a new type of zinc-based aluminum rich alloy composite material was prepared by SPS spark plasma sintering technology. The microscopic morphology, element distribution, phase composition, interface structure, hardness and friction and wear properties of metallized carbon fiber and composite materials have been systematically studied, it provides a theoretical reference for the preparation of carbon fiber reinforced zinc-based aluminum rich alloy composites with excellent friction and wear properties.

## 2. Experimental Materials and Methods

### 2.1. Experimental Materials

The chemical composition of the new zinc-based aluminum rich alloy composite material and ZA27 alloy material developed in this experiment are shown in Table 1. The metal powder used in the experiment is chemically pure. The particle size of the ZA27 alloy powder is 300 meshes. The TC-35 type 12K carbon fiber is selected as the research object. The basic performance parameters are shown in Table 2. The chopped carbon fiber length is 0.2 mm.

### 2.2. Experimental Method

**Figure 1:**
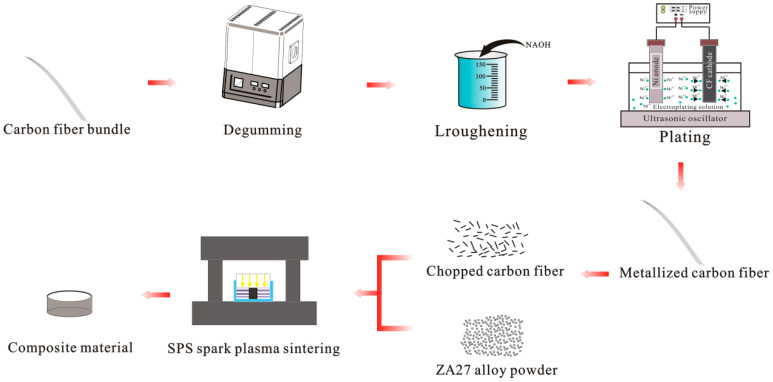
Composite material preparation process.

Due to the poor interfacial bonding between carbon fiber and ZA27 alloy, a nickel layer was plated on the surface of the carbon fiber by electroplating to improve the interfacial bonding between the carbon fiber and the matrix material. The carbon fiber reinforced zinc-based aluminum rich alloy was prepared by SPS spark plasma sintering. For composite materials, the experimental process is shown in Figure 1.

#### 2.2.1. Pretreatment Process

Surface pretreatment is required before metallization of the carbon fiber surface. First, degumming (hot degreasing + acetone solution immersion): wash the crucible clean with deionized water, dry it in a drying furnace, take it out and add a weighing scale to the crucible put the measured carbon fiber bundle into a DRZ resistance furnace, heat it up to 400 ℃ and burn it for 120 min to burn off the organic binder on the surface, soak it in an acetone solution for 60 min to dissolve the coke, and wash it with deionized water. After being dried, it is ready for use. Its purpose is to remove the epoxy resin covering the surface of commercial carbon fiber. Then roughen the surface: place the degummed carbon fiber bundle in NaOH solution to roughen it, while rotating it on a smart magnetic stirrer and heating it for 15 min, then take out the carbon fiber bundle and rinse it with deionized water until it is neutral, and put it in electric heating drying in a constant temperature drying box in order to increase the surface roughness of the fiber and the polar group, thereby increasing the surface activity, improving the affinity with the metal base, and prepare for the metallization of the carbon fiber surface.

#### 2.2.2. Metallization of Carbon Fiber Surface

**Figure 2:**
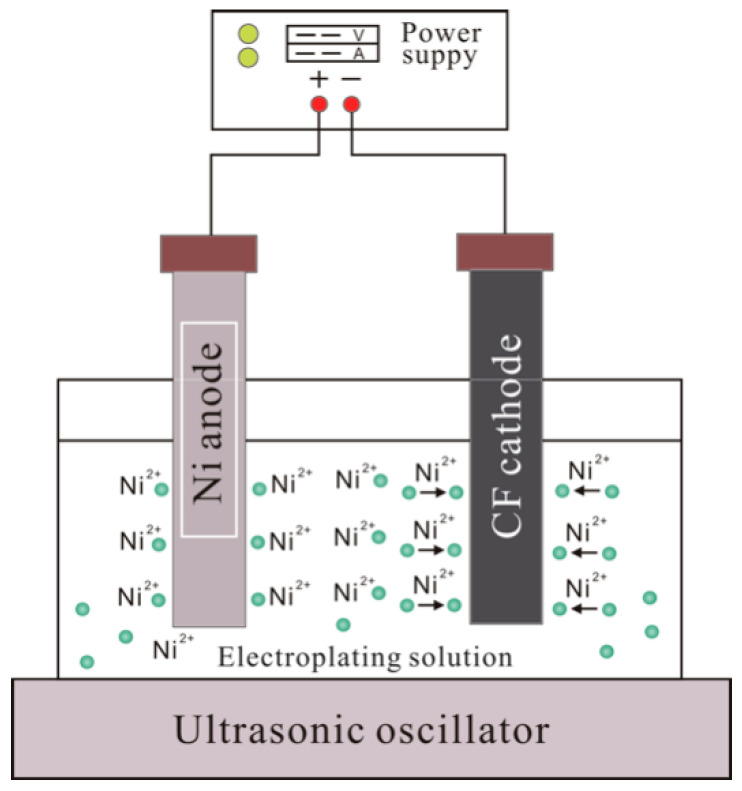
Electroplating device.

The carbon fiber bundle after the above pretreatment is placed in an electroplating device for electroplating. The composition and process parameters of the electroplating solution are shown in Table 3 and Table 4. The steps of nickel electroplating in this experiment are as follows: Step 1, weigh the required nickel sulfate, nickel chloride, boric acid and sodium dodecyl sulfate, and dissolve them in an appropriate amount of deionized water, mix them in sequence, and then mix them. Add hydrochloric acid dropwise to the plating solution, adjust the pH of the plating solution to 3, and dilute the volume to 500 mL; Step 2, use sandpaper to remove the oxides on the surface of the high-purity nickel plate, ultrasonically clean for 30 min, and rinse with deionized water; Step 3, the carbon fiber bundle and the high-purity nickel plate are, respectively, fixed to the cathode and anode of the power supply, and placed in the electroplating device as shown in Figure 2, electroplating is carried out by controlling the current, voltage and electroplating time, and then washed with deionized water and dried.

#### 2.2.3. Blending and SPS Spark Plasma Sintering

**Figure 3:**
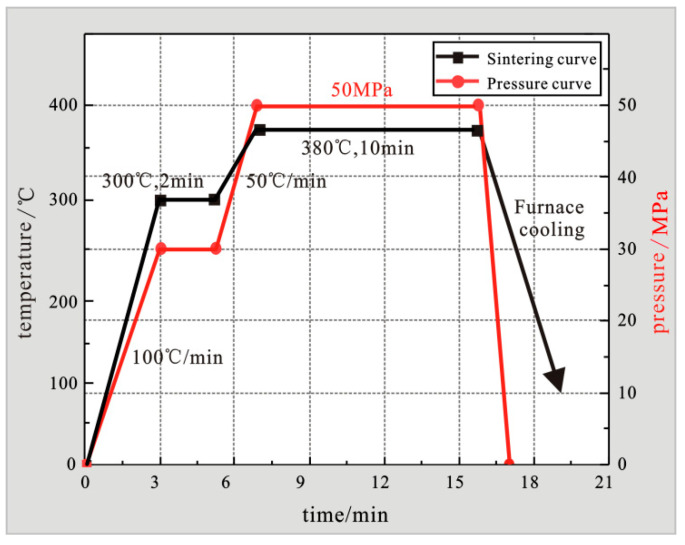
Process curve diagram of composite materials prepared by SPS spark plasma sintering method.

Use a carbon fiber cutter to cut the metalized carbon fiber bundles into 0.2 mm chopped carbon fibers. Use a V-type blender to evenly disperse the chopped carbon fibers in the matrix powder. Put the mixture into a graphite mold and placed in SPS spark plasma sintering equipment for sintering. Firstly, by adjusting the sintering time of the composite material for 3 min, the sintering temperature is increased from room temperature to 300 °C at 100 °C/min, and the pre-pressurization is 15 kN, and the pressure is maintained for 2 min. Secondly, increase the sintering temperature from 300 °C to 380 °C at a heating rate of 50 °C/min, hold for 10 min, and increase the sintering pressure to 30 kN, and then cool down with the furnace to obtain a carbon fiber reinforced zinc-based aluminum rich alloy composite material. In addition, during the sintering process, the sample is fully loaded to 50 MPa, and the specific SPS spark plasma sintering process curve is shown in Figure 3.

#### 2.2.4. Compound Representation

In this experiment, the ZEISS Gemini 300 scanning electron microscope was used to observe and analyze the surface morphology and cross-sectional morphology of the carbon fiber before and after the nickel coating, and the carbon fiber reinforced zinc-based aluminum rich alloy composite material was mapped and scanned to obtain the element distribution of the composite material figure, characterize the phase of the composite material with an x-ray diffractometer. At the same time, the HVS-1000Z digital micro-hardness tester (HV0.1 = 100 MPa, 10 s) is used to test the hardness of the composite material. A sample with a thickness of 7 mm and a diameter of 30 mm is ground and polished to obtain a smooth and flat surface. The test is carried out with a diamond spherical indenter, the test force is 9.807 N, the holding time is 15 s, 6 points are selected for each sample to be tested and the average value is taken. The dry sliding friction and wear test was carried out by the pin-disc wear tester UMT-3. The friction pair was a GCr15 alloy steel ball with a diameter of 6 mm. Before the test, the two parallel surfaces of the sample were polished and polished with 2000 grit sandpaper. The following applications were applied test parameters: At room temperature, the applied load is 8 N, the friction stroke is 5 mm, the friction frequency is 2 Hz, and the wear test cycle is 30 min. After the sliding test, each sample was cleaned with ethanol to remove potential contaminants, and the wear surface and wear volume of the sample were characterized by scanning electron microscope and MicroXAM-800 non-contact optical profiler.

## 3. Results and Discussion

### 3.1. The Microscopic Morphology and Phase Analysis of the Metallization of the Carbon Fiber Surface

**Figure 4:**
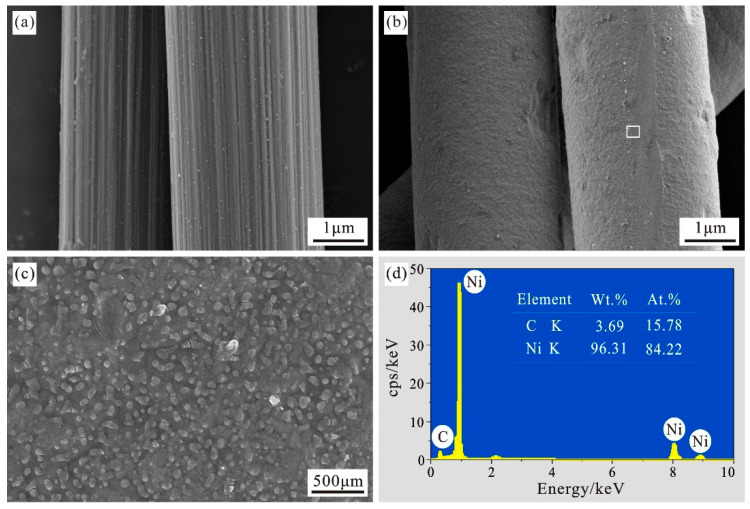
Surface morphologies of carbon fibers before and after nickel plating. (**a**) Original state; (**b**) After nickel plating; (**c**) High magnification image of the selected area in (**b**); (**d**) EDS spectrum of nickel-coated carbon fiber.

Figure 4a is the surface morphology of carbon fiber before electroplating. It can be clearly seen that there is no colloidal substance on the surface of carbon fiber after being roughened by NaOH solution, and there are obvious longitudinal grooves, and the surface roughness has increased. At the same time, the surface of carbon fiber has no obvious damages such as splits and fractures, and longitudinal grooves can increase the contact area and interface bonding force between the carbon fiber and the nickel layer. Figure 4b shows the micro-morphology of the nickel-plated carbon fiber surface at a bath temperature of 25 °C, a plating time of 20 min, a current of 2.3 A, and a voltage of 0.05 V. It can be seen that the nickel layer completely covers the carbon fiber, and the surface is plated. The particles of the nickel layer are dense and uniform as a whole, and there is no obvious drop-off and other defects. According to the observation in Figure 4c, there are some round cell-shaped protrusions on the plating layer, which are caused by the growth method of the nickel layer. It is a relatively complicated process involving the nucleation and nucleation of crystals and the catalytic deposition of nickel particles. When electroplating has just been carried out, the bottom of the groove on the carbon fiber surface is likely to preferentially deposit nickel particles to form nickel nuclei. As the electroplating process progresses, the carbon fiber protrusions and groove sides that are not conducive to nucleation will also be nickel nuclei appear, and then over time the entire carbon fiber surface will be covered with nickel nuclei and grow up at the same time. As the electroplating time increases, the crystal nuclei initially formed at the bottom of the groove on the surface of the carbon fiber and the incomplete structure gradually grow up, and the small particle nuclei continue to gather into large particles, and finally form a continuous and uniform nickel layer. Furthermore, a round cell-shaped protrusion is formed on the surface [25]. These protrusions had increased the surface roughness of the fiber, which is conducive to the combination of the fiber and the aluminum matrix. EDS quantitative analysis of part of the coating in the frame of Figure 4b obtains the weight percentage and atomic percentage of the elements. From the energy spectrum of Figure 4d and the data in the table, it can be seen that the coating contains nickel and carbon. It shows that there aren’t any new impurity elements are involved in the electroplating process, and the spectral peak corresponding to nickel is the highest. The weight percentages of nickel and carbon are 96.31% and 3.69%, and the atomic content is 84.22% and 15.78%, respectively.

**Figure 5:**
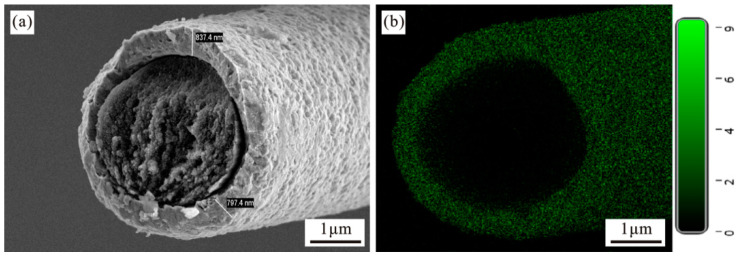
Cross-sectional topography of nickel-coated carbon fiber. (**a**) Cross-section of nickel-coated carbon fiber; (**b**) Scanning image of nickel-coated carbon fiber.

Figure 5a is a cross-sectional morphology of nickel-plated carbon fiber. It can be seen that the plated nickel layer is evenly distributed and surrounds the carbon fiber, and it is tightly attached to the surface of the carbon fiber. The average thickness of the nickel layer is about 817.4 nm. Since the left side of the carbon fiber is close to the anode nickel plate, and the right side is farther from the anode nickel plate. Thus, the atom movement distance is too far, which means it cannot be attached to the right side of the carbon fiber in time, which may result in uneven coating thickness [26]. Figure 5b is a scan of the plated carbon fiber. It is observed that the nickel element is uniformly distributed on the surface of the carbon fiber, indicating that the nickel-plating layer completely wraps the carbon fiber and forms the structure of the shell.

### 3.2. Microscopic Morphology and Phase Analysis of Carbon Fiber Reinforced Zinc-Based Aluminum Rich Alloy Composites

**Figure 6:**
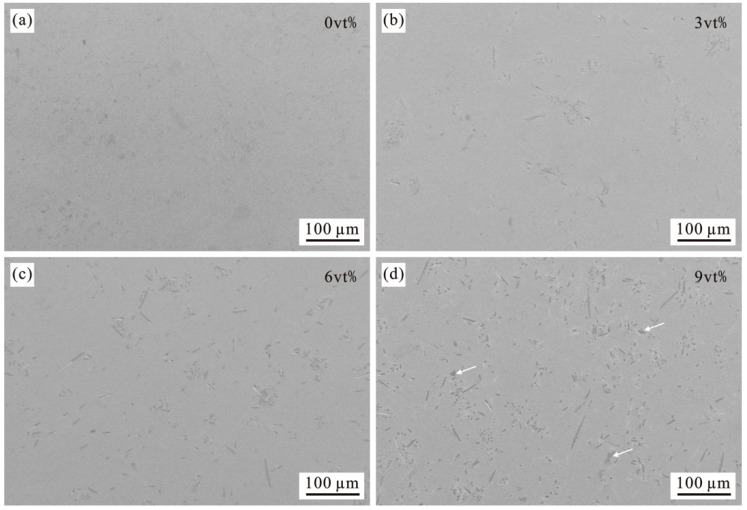
Distribution of carbon fibers in composites. (**a**) 0vt% CF; (**b**) 3vt% CF; (**c**) 6vt% CF; (**d**) 9vt% CF.

Figure 6 is the actual distribution of carbon fiber content of 0vt%, 3vt%, 6vt%, and 9vt% in the composite material under the scanning electron microscope. It can be seen from the above figures that the carbon fiber in the composite material is basically evenly distributed, and the overall carbon fiber is short in the rod shape, some of the carbon fibers are concentrated and then appear as dots. The nickel-plated carbon fibers may be broken due to the different arrangement of the carbon fibers or the high pressure during the pressing process. The high content of carbon fiber with a volume fraction of 9vt% leads to obvious local agglomeration. The agglomeration of carbon fiber blocks the diffusion of the matrix material, which has caused the carbon fiber and the matrix material could not be tightly combined during the sintering process. Therefore, the holes have occurred.

**Figure 7:**
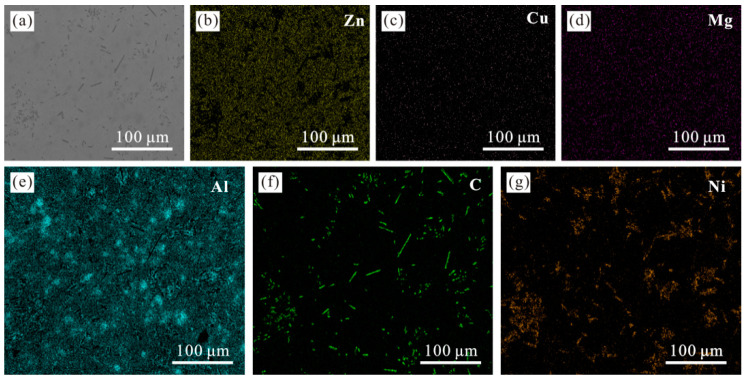
Surface morphology and element distribution of composites. (**a**) Surface morphology of composites; (**b**) Distribution of zinc; (**c**) Distribution of copper; (**d**) Distribution of magnesium; (**e**) Distribution of aluminum; (**f**) Carbon distribution; (**g**) Nickel distribution.

For 6vt% composites of carbon fiber content mapping scanning, composite material distribution of each element, from Figure 7b–e it can be seen that carbon fiber reinforced zinc-based aluminum rich alloy composite material of zinc, copper, magnesium, aluminum four elements distribution is relatively uniform. It can be seen in Figure 7f,g, carbon and nickel element has a concentration and whole are dispersed in the matrix, and to distinguish clearly, it shows that the surface plating nickel short carbon fiber dispersed uniformly in the matrix, the basic no reunion phenomenon, while the distribution of carbon and nickel elements are basically identical, but the part of the nickel elements have obvious diffusion phenomenon, in sintering process of nickel coating on the surface of the carbon fiber, may spread to the substrate [27].

**Figure 8:**
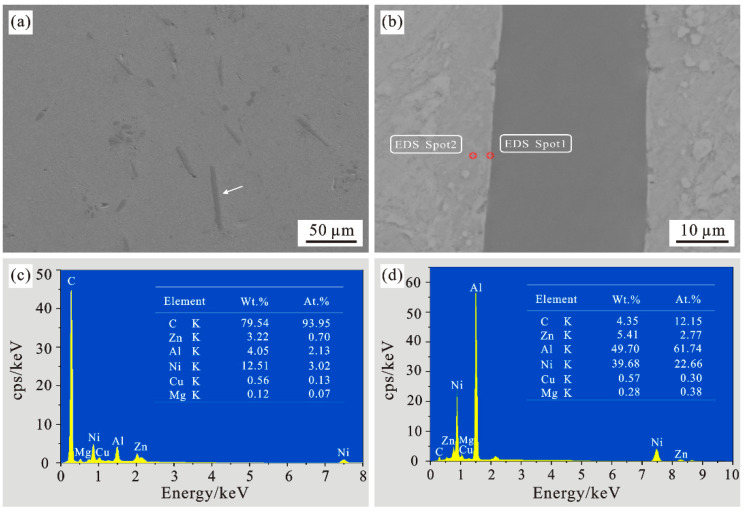
Microscopic topography of the composite material. (**a**) Microscopic topography of the composite material; (**b**) High magnification image of the specified area in (**a**); (**c**) EDS pattern at spot 1; (**d**) EDS pattern at spot 2.

**Figure 9:**
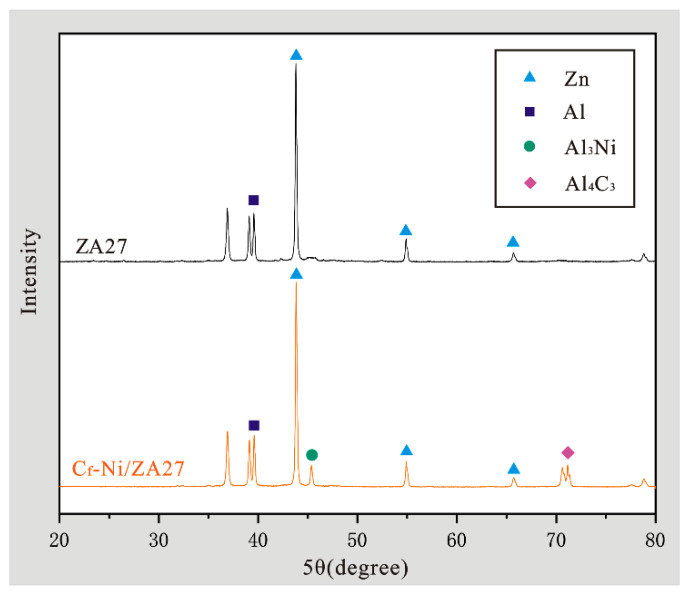
XRD analysis diagram of composite materials.

Figure 8 is a microscopic morphology of the composite material with a carbon fiber content of 6vt%. It can be seen from Figure 8a that the material is tightly integrated without obvious pores. Figure 8b shows that the nickel-plated short carbon fiber and the matrix material is tightly bonded, and the matrix material is completely covered on the surface of the nickel-plated carbon fiber, forming a dense microstructure, and no obvious pores or carbon fiber damage are observed. The nickel-plating layer is highly reliable and it has excellent oxidation resistance, so when it is compounded with the matrix material at high temperatures, it can effectively prevent the carbon fibers from being damaged, and prevent the direct contact and agglomeration between the carbon fibers. However, there is no clear nickel-plating profile in Figure 8b which means that the nickel plating around the carbon fiber may partially disappear. It can be verified from the scanning results of Figure 8c,d, in Figure 8b spot1 it can be seen in Figure 8c that most of the elements in the bonding site are carbon elements, and contain less nickel, zinc and aluminum. Compare the scan results of spot2 in Figure 8b–d, it is found that the nickel content in the area far from the joint is significantly increased compared to the joint. The weight percentage of nickel increases from 12.51% at spot1 to 39.68% at spot2, indicating that the nickel-plating layer is gradually moving towards the substrate during the sintering process. In the diffusion, and the formation of intermetallic compounds. Figure 9 shows the XRD diffraction pattern of the composite material, compared with ZA27, it can be seen that the Ni coating on the carbon fiber surface in the composite material gradually diffuses into the matrix, and reacts with ZA27 to form Al_3_Ni phase, which is distributed near the carbon fiber, the Al_3_Ni phase is generated with the carbon fiber as the nucleation center and grows at the interface of the carbon fiber. At the interface between the carbon fiber and the substrate, a nickel-rich area is formed in the contact area with the coating, and a nickel-poor area is formed in the area away from the coating. Such a concentration gradient change can indicate that the nickel coating has diffused. In the XRD diffraction pattern detection results of Figure 9, in addition to the reaction of Ni coating and ZA27 to form Al_3_Ni phase, a small amount of Al_4_C_3_, brittle phase is formed between ZA27 and carbon fiber, which may be due to the fact that both ends of chopped carbon fiber are not covered by nickel layer and the matrix is in contact Generated, At the same time, in Figure 8b, the carbon content at spot2 is significantly lower than that at spot1, and its weight percentage decreases from 79.54% at spot1 to 4.35% at spot2, indicating that the intermetallic compound formed between the nickel coating and the substrate can It can effectively inhibit the diffusion of carbon fibers during the sintering process, thereby inhibiting the formation of a large number of brittle carbides Al_4_C_3_, and improving the interface bonding between carbon fibers and matrix materials.

### 3.3. Performance Analysis

#### 3.3.1. Hardness of Composite Materials

**Figure 10:**
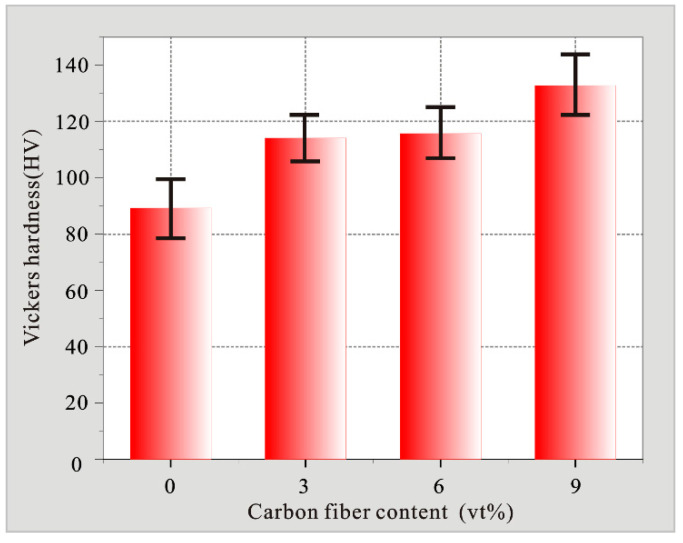
Hardness change diagram of different content of carbon fiber content.

At least 6 hardness measurements were performed on different positions of each sample, and the average of these measurements was reported as the Vickers hardness of the sample. As shown in Figure 10, the ZA27 matrix sample has the lowest hardness of 89.22 HV among all samples. The hardness of the sample increases with the increase of carbon fiber content. The average hardness is 114.11 HV, 115.715 HV, 132.766 HV. It can be observed that the hardness of ZA27 can be increased to 27.9% by incorporating 3vt% carbon fiber, 6vt% carbon fiber can be increased to 29.6%, and 9vt% carbon fiber can be incorporated. It can be increased to 49.4%. The increase in hardness is due to the fact that the carbon fiber itself is harder than the matrix material, and at the same time, the carbon fiber has a higher strength. The addition of ZA27 plays a role in the second phase strengthening. The increase in the hardness value of the composite material is also affected by the slightly increased carbide in these materials [28].

#### 3.3.2. Friction and Wear Properties of Composite Materials

**Figure 11:**
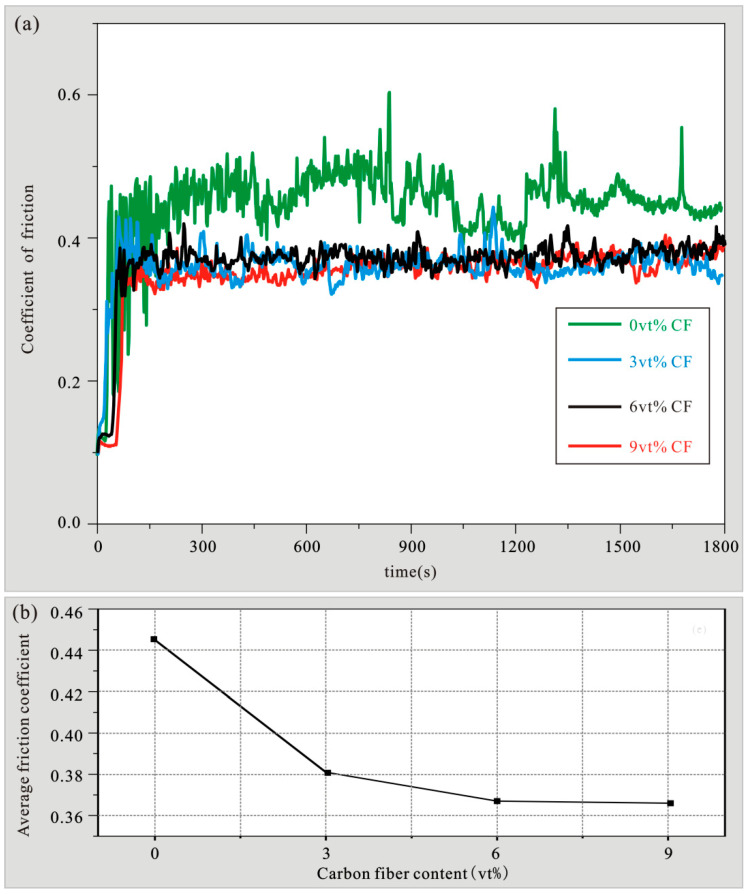
Variation diagram of friction coefficient of composite materials. (**a**) Dynamic variation of friction coefficient; (**b**) Variation trend of average friction coefficient.

**Figure 12:**
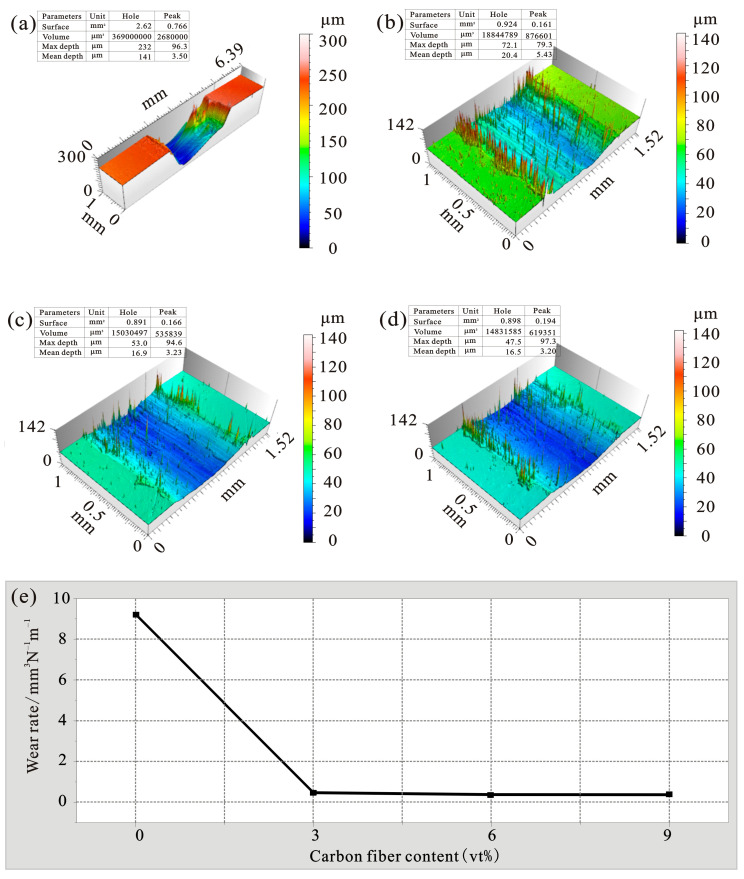
Changes of wear volume and wear rate of composites. (**a**) 0vt% CF; (**b**) 3vt% CF; (**c**) 6vt% CF; (**d**) 9vt% CF; (**e**) Change trend of wear rate.

**Figure 13:**
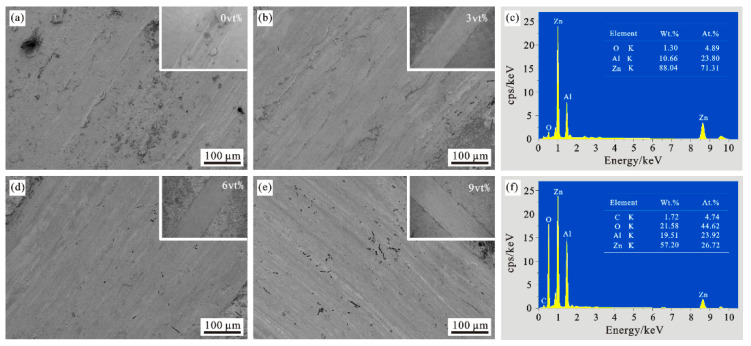
The worn surface topography of the composite material. (**a**) 0vt% CF; (**b**) 3vt% CF; (**c**) EDS spectrum of 0vt% CF composite;(**d**) 6vt%CF; (**e**) 9vt%CF; (**f**) EDS spectra of the 6vt%CF composite.

At room temperature, the test parameters are as follows: when the friction frequency is 2 Hz, the friction length is 5 mm, the applied load is 8 N, and the friction time is 30 min, the carbon fiber content increases to 0vt%, 3vt%, 6vt%, 9vt%, The obtained Figure 11 shows the change trend graph of the friction coefficient and the average friction coefficient of different content carbon fiber reinforced zinc-based aluminum rich alloy composites, and Figure 12 shows the three-dimensional profile of wear volume and the change trend graph of wear rate of different content carbon fiber reinforced zinc-based aluminum rich alloy composites. As can be seen from Figure 11, When the addition of carbon fiber is 0vt%, the average friction coefficient of the ZA27 alloy matrix is 0.445. With the increase of the carbon fiber content of the reinforcement, the friction coefficient of the composite material gradually decreases. When the addition of the reinforcement is 3vt%, the average friction coefficient drops to 0.38, which is 14.4% lower than the friction coefficient of the ZA27 alloy matrix. When the carbon fiber content is increased to 6vt%, the average friction coefficient is further reduced to 0.367, which is 18.4% lower than the friction coefficient of the ZA27 alloy matrix, and the carbon fiber content increases at 9vt%, the average friction coefficient is 0.366, which is basically as same as the friction coefficient when the content is 6vt%. Figure 12 shows the effect of carbon fiber volume fraction on the wear volume and wear rate of composite materials. Figure 12a–d shows that the addition of carbon fiber is 0vt%, 3vt%, 6vt%, 9vt% at this time, the wear volumes of the composite materials were 3.69 × 10^8^ μm^3^, 0.188 × 10^8^ μm^3^, 0.15 × 10^8^ μm^3^, and 0.148 × 10^8^ μm^3^. The wear rate is calculated by the following (1):

(1) $$\omega_{s}=\frac{v}{pl}$$

where *ω_s_* is the wear rate, *l* is the sliding distance, *v* is the wear volume of the sample, and *p* is the normal load. As the volume fraction of carbon fiber increases, the wear rate of the composite material decreases significantly. It is showed in Figure 12e. When the reinforcement addition is 0vt%, the wear rate of the ZA27 alloy matrix is 9.225 mm^3^N^−1^m^−1^, and when the reinforcement addition is 3vt%, the wear rate drops to 0.471 mm^3^N^−1^m^−1^, which is 95% lower than the wear rate of the alloy matrix. When the carbon fiber content is increased to 6vt%, the wear rate is further reduced to 0.376 mm^3^N^−1^m^−1^, which is 96% lower than the wear rate of the alloy matrix. The carbon fiber content when it is increased to 9vt%, the wear rate is 0.371 mm^3^N^−1^m^−1^. At this time, the wear rate remains basically unchanged with the increase of the carbon fiber content, and this trend is consistent with the change of the friction coefficient. From Figure 11a, it can be found that the friction coefficient of the ZA27 alloy substrate has obvious up and down fluctuations. This may be due to the mutual contact and friction between the surface of the ZA27 alloy material and the bearing steel GCr15 in the experiment, which has caused the surface of the substrate material to deform. Particles of different sizes are constantly falling off, which plays a part of the role of rolling friction between the friction pairs. The friction coefficient curves of 3vt%, 6vt%, and 9vt% in Figure 11a are relatively stable as a whole and are also significantly lower than the friction coefficient of the ZA27 alloy base material. This is due to the cyclic load generated during the wear process. The surface of the sample is plastically deformed. It can be seen from Figure 13a that the carbon fiber is exposed to the surface due to the removal of the surface layer. Under the action of the contact force, the carbon fiber is partially ground into fine carbon particles. As the volume fraction of carbon fibers in the composite increases, more carbon fibers are exposed to the surface, resulting in more carbon fibers being ground into fine carbon particles, a solid lubricant, which forms a thin film on the wear surface of the sample. Due to the existence of this film, it prevents the metal-to-metal contact between the composite material and its counterpart during the friction process, so that the friction coefficient of the composite material is reduced and reduced the wear rate of the composite material [29]. The enhancement of the friction and wear properties of composites is also affected by their hardness. In general, the harder the material, the better the friction and wear properties, because higher hardness materials are better at resisting plastic deformation or failure, resulting in more wear-resistant materials. The experimental results also show that adding carbon fiber can improve the hardness of ZA27 alloy, so the increased hardness by adding carbon fiber helps to improve the wear resistance of the composite material. The SEM micrograph and EDS spectrum of the wear surface of ZA27 alloy material and 6vt% carbon fiber reinforced zinc-based aluminum rich alloy composite material is shown in Figure 13. The wear surface of ZA27 alloy material is mainly composed of grooves and a large amount of wear debris, as shown in Figure 13a, the wear surface is damaged due to plastic deformation. Some of the sheared matrix material fragments are peeled off into flakes and ground and adhered to the surface of the material by the friction pair. The adhesion effect hinders the movement between the matrix material and the friction pair, adhesive wear has occurred with the base material. It can be seen from Figure 13c that the presence of oxygen on the worn surface also indicates that oxidative wear has occurred. As shown in Figure 13d, the wear surface of the 6vt% carbon fiber composite material has wider and shallower grooves than the ZA27 alloy material. The wear surface is smoother than the base material and there is no adhesion of debris. At the same time, due to shear the part where the force acts on the carbon fiber is also exposed on the surface of the material. Obviously, the addition of carbon fiber hinders the deformation of the matrix, which means that the wear rate is reduced. As shown in Figure 13f, the oxygen content on the surface is increased to 21.58wt% relative to the ZA27 alloy, and the carbon content is also increased to 1.72wt%. This indicates that there is a carbon-rich film in the area doped with carbon fiber on the surface of the 6vt% carbon fiber composite material. Since the carbon content is relatively low, the carbon film should only exist around the carbon fiber and not diffuse to the entire wear surface. At this time, the wear mechanism it is oxidative wear. As the load time continues to increase, the friction temperature also increases. If the heat generated by the friction cannot be dissipated in time, the temperature rise will change the surface properties of the friction contact surface. Another reason that carbon fiber reinforced zinc-based aluminum rich alloy composites exhibit better friction properties than ZA27 is to improve the thermal conductivity of the composites. Carbon fiber is an inorganic polymer fiber with a carbon content of more than 90%. The microstructure is similar to artificial graphite, it is a chaotic layer graphite structure, and its thermal conductivity is generally 0.025-0.09 W/(m·K). Due to its excellent thermal conductivity, the thermal conductivity of carbon fiber is higher than that of the alloy matrix. Therefore, the presence of carbon fiber can cause friction. The heat is dissipated in time to prevent the surface temperature from continuously increasing during the friction process, thereby optimizing the friction performance of the material.

## 4. Conclusions

(1) The nickel layer is plated on the surface of the carbon fiber by electroplating process. The nickel layer grows and distributes on the surface of the carbon fiber. The carbon fiber is completely covered without obvious fall off; Carbon fiber-reinforced zinc-based aluminum rich alloy composite materials with contents of 0vt%, 3vt%, 6vt%, and 9vt% were prepared by the SPS sintering method; nickel-plated carbon fiber is tightly combined with the matrix material and dispersed in the matrix material as a whole. Nickel plated carbon fiber is closely combined with the matrix material and dispersed into the matrix material as a whole. The intermetallic compound Al_3_Ni formed between the nickel coating and the matrix effectively inhibits the diffusion of carbon fiber in the sintering process, indicating that the nickel coating improves the interfacial adhesion between carbon fiber and matrix.
(2) The average hardness of carbon fiber composites with content of 0vt%, 3vt%, 6vt% and 9vt% are 89.22 HV, 114.11 HV, 115.72 HV and 132.77 HV, respectively. Compared with the ZA27 alloy matrix, 6vt% is added. The hardness of carbon fiber increased by 29.6%. With the increase of carbon fiber content, the friction coefficient and wear rate of the composite material gradually decrease. When the carbon fiber content increases to 6vt%, the friction coefficient and wear amount remain basically unchanged. At this time, the corresponding friction coefficient is 0.367 and the wear amount is 0.376 mm^3^N^−1^m^−1^, compared with the average coefficient of friction and wear rate of the matrix material, they are reduced by about 18.4% and 96%, respectively. These improvements can be attributed to the addition of carbon fiber, which enhances the hardness of the composite, improves the ability of the material to resist plastic deformation, reduces the metal-to-metal contact between the composite and its counterpart in the friction process, hinders the deformation of the matrix, and optimizes the friction and wear properties of the material.

## Figures and Tables

**Table 1:** Chemical composition of test materials.

Element	Al (wt%)	Mg (wt%)	Cu (wt%)	Cf (vt%)	Zn (wt%)
ZA27	27	0.04	2.5	0	Balance
Composite material 1	27	0.04	2.5	3	Balance
Composite material 2	27	0.04	2.5	6	Balance
Composite material 3	27	0.04	2.5	9	Balance

**Table 2:** Basic performance parameters of carbon fiber.

Grade	Number of Filaments/K	Tensile Strength/GPa	Tensile Modulus/GPa	Density/g·cm^3^	Monofilament Diameter/μm
TC-35	12	4.6	230	1.82	7.3

**Table 3:** Electroplating solution composition.

Element	Nickel Sulfate	Nickel Chloride	Boric Acid	Sodium Dodecyl Sulfate
concentration (g/L)	270	70	40	0.1

**Table 4:** Electroplating process parameters.

Process Parameters	Temperature (°C)	Time (min)	Current (A)	Voltage (V)	PH
	25	20	0.05	2.3	3

## Data Availability

Not applicable.

## References

[1] Wang Y.W., Zuo X.Q., Ran S.J. (2020). Effects of semi-solid extrusion and heat treatment on the microstructure, mechanics, and wear resistance of SiC/high aluminum zinc-base alloy composites. Mod. Phys. Lett. B.

[2] Krajewski W.K., Greer A.L., Bura J. (2019). New developments of high-zinc Al-Zn-Cu-Mn cast alloys. Mater. Today Proc..

[3] Mohammad M.K., Mehak N. (2022). Effect of in situ TiC reinforcement and applied load on the high-stress abrasive wear behaviour of zinc–aluminum alloy. Wear.

[4] Liu Y.X., Yin F.C., Li Z. (2020). Effects of adding aluminium in zinc bath on Co-Zn interfacial reaction. Indian J. Eng. Mater. Sci..

[5] Altuncu E., Iric S. (2017). Evaluation of fracture toughness of thermal sprayed and hard chrome coated aluminium-zinc alloy. Acta Phys. Pol. A.

[6] Bansod A.V., Patil A.P., Kalita K. (2020). Fuzzy multicriteria decision-making-based optimal Zn–Al alloy selection in corrosive environment. Int. J. Mater. Res..

[7] Lu X.Q., Wang S.R., Xiong T.Y. (2019). Study on corrosion resistance and wear resistance of Zn-Al-Mg/ZnO composite coating prepared by cold spraying. Coatings.

[8] Bican O., Savaskan T. (2014). A comparative study of lubricated friction and wear behaviour of Al–25Zn–3Cu–3Si bearing alloy. Proc. Inst. Mech. Eng. Part J J. Eng. Tribol..

[9] Li Q.L., Shi S.M., Li X. (2020). Study on low velocity cyclic impact wear of amorphous carbon films with different mechanical properties. Surf. Coat. Technol..

[10] Mao G., Liu D., Gao W. (2021). The effects of copper (Cu) or zinc (Zn) on fluidity of A357 alloy. Mater. Lett..

[11] Miloradovi N., Vujanac R., Pavlovi A. (2020). Wear Behaviour of ZA27/SiC/Graphite Composites under Lubricated Sliding Conditions. Materials.

[12] Yu Z.D., Chen M.H., Li F.J. (2020). Synergistic effect of corrosion and wear of the 316 stainless steel in molten zinc alloy at 460 degrees C. Corros. Sci..

[13] Kiran T.S., Kumar M.P. (2013). Mechanical properties of as-cast ZA-27/Gr/SiCp hybrid composite for the application of journal bearing. J. Eng. Sci. Technol..

[14] Sharma S.P., Ting J.M., Vilar R. (2020). Electron Microscopy Study of Surface-Treated Carbon Fiber for Interface Modification in Composites. Diam. Relat. Mater..

[15] Zhou L.L., Li X.W., He D.Y., Guo W.L., Huang Y.F., He G.C., Xing Z.G., Wang H.D. (2022). Study on Properties of Potassium Sodium Niobate Coating Prepared by High Efficiency Supersonic Plasma Spraying. Actuators.

[16] Li X.W., Yu Q.Y., Chen X., Zhang Q.X. (2021). Microstructures and electrochemical behaviors of casting magnesium alloys with enhanced compression strengths and decomposition rates. J. Magnes. Alloys.

[17] Jia Y., Ajayi T.D., Wahls B.H. (2020). Multifunctional Ceramic Composite System for Simultaneous Thermal Protection and Electromagnetic Interference Shielding for Carbon Fiber-Reinforced Polymer Composites. ACS Appl. Mater. Interfaces.

[18] Jia Y., Ajayi T.D., Ramakrishnan K.R. (2020). A skin layer made of cured polysilazane and yttria stabilized zirconia for enhanced thermal protection of carbon fiber reinforced polymers (CFRPs). Surf. Coat. Technol..

[19] Zou Y., Li C.H., Hu L. (2020). Effects of short carbon fiber on the macro-properties, mechanical performance and microstructure of SiSiC composite fabricated by selective laser sintering. Ceram. Int..

[20] Mahaviradhan N., Sivaganesan S., Sravya N.P. (2020). Experimental study on mechanical properties of carbon fiber reinforced aluminum metal matrix composites. Mater. Today.

[21] Sha J.J., Zhao-Zhao L., Sha R.Y. (2021). Improved wettability and mechanical properties of metal coated carbon fiber-reinforced aluminum matrix composites by squeeze melt infiltration technique. Trans. Nonferrous Met. Soc. China.

[22] Song Y., Fan J.L., Wu S. (2019). Effect of carbon-fibre powder on friction and wear properties of copper-matrix composites. Met. Sci. J..

[23] Lin L., Schlarb A.K. (2019). Recycled carbon fibers as reinforcements for hybrid PEEK composites with excellent friction and wear performance. Wear.

[24] Ma X., Sun H., Kou S. (2021). Flexural strength and wear resistance of C/C-SiC brake materials improved by introducing SiC ceramics into carbon fiber bundles. Ceram. Int..

[25] Han W., Qian X., Ma H. (2021). Effect of nickel electroplating followed by a further copper electroplating on the micro-structure and mechanical properties of high modulus carbon fibers. Mater. Today Commun..

[26] Zhang J., Liu S., Liu J. (2019). Electroless nickel plating and spontaneous infiltration behavior of woven carbon fibers. Mater. Des..

[27] Zhu C., Su Y., Zhang D. (2020). Effect of Al_2_O_3_ coating thickness on microstructural characterization and mechanical properties of continuous carbon fiber reinforced aluminum matrix composites. Mater. Sci. Eng. A.

[28] Meng X., Choi Y., Matsugi K. (2018). Microstructures of Carbon Fiber and Hybrid Carbon Fiber-Carbon Nanofiber Reinforced Aluminum Matrix Composites by Low Pressure Infiltration Process and Their Properties. Mater. Trans..

[29] Lin Y., Zia A.W., Zhou Z. (2017). Development of diamond-like carbon (DLC) coatings with alternate soft and hard multilayer architecture for enhancing wear performance at high contact stress. Surf. Coat. Technol..

